# Molecular Diversity and Population Structure of a Worldwide Collection of Cultivated Tetraploid Alfalfa (*Medicago sativa* subsp. *sativa* L.) Germplasm as Revealed by Microsatellite Markers

**DOI:** 10.1371/journal.pone.0124592

**Published:** 2015-04-22

**Authors:** Haiping Qiang, Zhihong Chen, Zhengli Zhang, Xuemin Wang, Hongwen Gao, Zan Wang

**Affiliations:** 1 Institute of Animal Sciences, Chinese Academy of Agriculture Sciences (CAAS), Beijing 100193, China; 2 National Animal Husbandry Service, Ministry of Agriculture, Beijing 100125, China; Chinese Academy of Sciences, CHINA

## Abstract

Information on genetic diversity and population structure of a tetraploid alfalfa collection might be valuable in effective use of the genetic resources. A set of 336 worldwide genotypes of tetraploid alfalfa (*Medicago sativa* subsp. *sativa* L.) was genotyped using 85 genome-wide distributed SSR markers to reveal the genetic diversity and population structure in the alfalfa. Genetic diversity analysis identified a total of 1056 alleles across 85 marker loci. The average expected heterozygosity and polymorphism information content values were 0.677 and 0.638, respectively, showing high levels of genetic diversity in the cultivated tetraploid alfalfa germplasm. Comparison of genetic characteristics across chromosomes indicated regions of chromosomes 2 and 3 had the highest genetic diversity. A higher genetic diversity was detected in alfalfa landraces than that of wild materials and cultivars. Two populations were identified by the model-based population structure, principal coordinate and neighbor-joining analyses, corresponding to China and other parts of the world. However, lack of strictly correlation between clustering and geographic origins suggested extensive germplasm exchanges of alfalfa germplasm across diverse geographic regions. The quantitative analysis of the genetic diversity and population structure in this study could be useful for genetic and genomic analysis and utilization of the genetic variation in alfalfa breeding.

## Introduction

Cultivated tetraploid alfalfa (*Medicago sativa* subsp. *sativa* L.), a perennial autotetraploid (2n = 4x = 32) and cross-pollinated forage legume, is the most important cultivated forage plant in the world [1]. In the last century, many traits such as disease resistance, insect resistance, winter survival, etc., had been successfully improved by phenotypic selection protocols in alfalfa. Unfortunately, improvement in the yield of alfalfa has been stagnated in recent years [2–3]. Association mapping using diverse genotypes in plants is a new and powerful tool that has begun to yield promising results in identifying the functional variation in both known and unknown genes associated with important agronomic and economic traits [4–5]. Genetic diversity, population structure, and linkage disequilibrium (LD) of a population provide strategic information for association mapping and marker-assisted breeding [5]. Estimation of population diversity and structure of germplasm have been carried out in many plant species, including wheat (*Triticum aestivum* L.) [6], rice (*Oryza sativa* L.) [7], perennial ryegrass (*Lolium perenne* L.) [8], foxtail [*Setaria italica* (L.) Beauv.] [9–10], cucumber (*Cucumis sativu*s L.) [11], and cotton (*Gossypium hirsutum* L.) [12]. The genetic diversity of alfalfa was well assayed by different molecular marker systems [13–16]. Tucak et al. [17] assessed the efficiency of phenotypic and DNA markers for genetic diversity of ten alfalfa accessions and concluded that molecular markers might be useful for grouping of germplasm with similar genetic background. Sakiroglu et al. [18] estimated genetic diversity and determined population structure in a collection of unimproved diploid accessions with 89 polymorphic simple sequence repeat (SSR) markers. Li et al [19] investigated population structure in a tetraploid alfalfa breeding population using genome-wide SSR markers. No obvious population structure was found in the alfalfa breeding population, which could be due to the relatively narrow genetic base of the founders and/or due to two generations of random mating [19].

Although several studies have employed marker-based estimation of genetic diversity and population structure in alfalfa, most of these studies are limited in the number of accessions included or the number of markers used to characterize genetic diversity in alfalfa. Some of these studies have been conducted using germplasm specific for a breeding program [19], or diploid unimproved alfalfa germplasm [18]. Therefore, a comprehensive study involving a worldwide collection of cultivated tetraploid alfalfa germplasm is still needed to quantify overall genetic diversity in alfalfa for its effective utilization in breeding, genetic, and genomics studies in alfalfa. Accordingly, the objectives of this study were to estimate the genetic diversity, and population structure of a worldwide collection of cultivated tetraploid alfalfa.

## Materials and Methods

### Plant materials

A total of 336 cultivated tetraploid genotypes from 75 *M*. *sativa* subsp. *sativa* accessions were analyzed in the study. Each accession was represented by four genotypes, except for the Chinese accessions that were represented by eight genotypes for each accession. Nine accessions from China were obtained from National Herbage Germplasm Bank of China; two accessions from Syria, one from Libya and one accession from Sudan were provided by the Institute of Animal Science, Chinese Academy of Agricultural Science (Beijing, China); the other 62 accessions were provided by the USDA National Plant Germplasm System (NPGS). Detailed information about the 336 genotypes used in this study is provided in S1 Table.

### SSR genotyping

Young leaves of each of all 336 genotypes were freeze-dried and ground to a fine powder, separately. Genomic DNAs were extracted from each powder sample, following the CTAB method [20]. DNA quality was tested using 1% agarose gel electrophoresis. The working concentration was adjusted to approximately 50ng/μL. In a preliminary study, we used a panel of eight genotypes to identify SSR markers that gave reproducible amplification and could be confidently scored. Out of 175 SSR primer pairs initially tested, 159 were selected for genotyping the whole panel of 336 samples. These selected markers covered all eight chromosomes of alfalfa genome, with a minimum of four markers per chromosome. Primer sequences for all SSR markers are publically available and were obtained from the previous publications [21–22]. A fluorescent 6-FAM or HEX labeled SSR primers was separately added to the PCR mix to generate fluorescent-labeled amplified products. PCR amplifications of genomic DNA was carried out in a 25μL reaction volume in an authorized Thermal Cycler (BBI, Canada) containing 2.5μL 10×buffer (100 mM Tris-HCl, pH 8.8 at 25°C; 500 mM KCl, 0.8%(v/v) Nonidet), 0.5μL 10mM dNTPs, 0.2μL(5U/μL) TaqDNA polymerase, 0.5μL(μmol/L) of each primer, 2μL25mM MgCl_2_, 1μL template DNA, 17.8μL ddH2O. The following PCR program was used: an initial denaturing for 3min at 95°C, followed by 10 cycles of 95°C for 30sec, 60°C for 30sec, 72°C for 30sec; and 20 cycles of 95°C for 30sec, 55°C for 30sec, 72°C for 30sec; a final extension at 72°C for 6min. PCR products were separated on an ABI3730xl DNA Analyzer (Applied Biosystems, Foster City, CA, USA). Fluorescence-labeled primers were synthesized at Applied Biosystems Company. Fragment sizes were determined using an internal size standard (LIZ500, ABI, USA), and the outputs were analyzed using GeneMapper software (http://www.appliedbiosystems.com.cn/).

### Data analysis of genetic diversity and population structure

Individual tetraploid genotypes were scored from microsatellite banding patterns in the electropherograms following the Microsatellite DNA Allele Counting-Peak Ratios (MAC-PR) method of Esselink et al [23]. For each locus, all alleles were analyzed in pairwise combinations to determine their dosages in the individuals samples by calculating the ratios between peak areas for all allele-pairs that were amplified simultaneously (see Esselink et al [23] for a full description of the procedure). Allele frequencies, expected heterozygosity (He) were calculated using AUTOTET [24]. The polymorphism information content (PIC) of SSR markers was calculated by PIC_CALC0.6 (http://hi.baidu.com/luansheng1229/item/306815126d58e3a4feded5a4) described by Botstein et al. [25]. The significant tests of genetic diversity between chromosomes, subpopulations and improvement status were conducted according to LSD method using SAS 8.2 (SAS, Cary, NC). The STRUCTURE software was used to infer the population structure of the entire set of genotypes [26]. Admixture model was used with the option of correlated allele frequencies between populations. Ten runs were conducted for each value of number of populations (K), with K ranging from 1 to 10. The length of burn-in Markov Chain Monte Carlo (MCMC) replications was set to 10,000 and data were collected over 100,000 MCMC replications in each run. We identified the optimal value of K using both the ad hoc procedure described by Pritchard et al. [26] and the method developed by Evanno et al. [27] with the help of Structure Harvester software [28]. The POPULATIONS version 1.2.28 (O. Langella 1999 unpublished, http://www.pge.cnrs-gif.fr/bioinfo/populations/index.php) was used to calculate pairwise genetic distances between the genotypes using Nei et al.’s [29] DA distance. The distance matrix was used to construct a dendrogram using neighbor joining method in MEGA 4[30]. Further analysis of genetic structure was done by Principal co-ordinate analysis (PCoA) using GenAlEx [31] and the tri-scatter plot was generated using the SAS 8.2 software [32]. We used Analysis of Molecular Variance (AMOVA) to partition molecular genetic variance within and among five subpopulations suggested by our Structure analysis. We conducted the AMOVA using the software program GenAlEx 6.1[31].

## Results

### Profiling of SSR markers in tetraploid alfalfa

Out of the 159 SSR primer pairs used for genotyping, 74 could not be scored with confidence. The remaining 85 markers amplified a total of 1056 alleles, confirming that most of the selected markers were highly informative. The average allele number per locus was 12.4, ranging from 4 to 40. The expected heterozygosity (He) ranged from 0.283 to 0.901 with the average of 0.677 and the polymorphism information content (PIC) ranged from 0.273 to 0.893 with an average of 0.638. A summary of genetic characteristics of 336 alfalfa genotypes based on 85 SSR markers are listed in S2 Table. The marker mtic238, located on chromosome 5, had the highest number of alleles (40), while bg183, aw317, mtic470, aw352, and bi112 each had only four alleles. Bf111 and bf644149, both located on the chromosome 2, shared the highest and lowest genetic diversity respectively, with the value of 0.901 and 0.283 in the He while 0.893 and 0.273 in PIC (S2 Table). Out of 1056 alleles detected, 172 were unique alleles (for those only found once in one accession), accounting for 16.3%. Five hundred and forty-eight were rare alleles which frequencies was less than 5%, accounting for 51.9%.

A comparative analysis of genetic characteristics was performed at the chromosome level (Table 1). The mean allele number per chromosome ranged from 8.7 for chromosome 6 to 15.6 for chromosome 2 (Table 1). Different chromosomes exhibited different allele number distributions. A large proportion of markers of chromosomes with allele number below nine (ca 64.2%) was observed while chromosomes 2 and 3 were characterized by the high proportion of markers with an allele number above nine (ca. 77% and 88%, respectively). Violin plots showing the distribution of allele numbers by chromosome and chromosome specific averages are shown in S1A Fig. The mean He values varied from 0.629 for chromosome 8 to 0.762 for chromosome 2 (Table 1). Markers from chromosomes 2 and 3 exhibited a large proportion of markers with mean He values above 0.7 (ca. 89%) (S1B Fig). The mean PIC values ranged from 0.591 for chromosome 8 to 0.736 for chromosome 2 (Table 1). The PIC values distribution patterns revealed by the markers from individual chromosomes were similar to the mean He (S1C Fig). Therefore, chromosomes 2 and 3 had the highest genetic diversity which revealed by the allele number, He and PIC (Table 1).

**Table 1:** Summary of genetic diversity at chromosome level.

Chromosome	No. of markers	Mean of alleles	Expected heterozygosity	PIC
1	19	11.3(±4.51)^a^	0.639(±0.106)^a^	0.593(±0.108)^a^
2	9	15.6(±7.23)^a^	0.762(±0.136)^a^	0.736(±0.140)^a^
3	9	14.7(±6.24)^a^	0.719(±0.184)^a^	0.694(±0.193)^a^
4	14	11.5(±7.53)^a^	0.669(±0.138)^a^	0.630(±0.148)^a^
5	9	12.9(±11.03)^a^	0.719(±0.116)^a^	0.681(±0.132)^a^
6	3	8.7(±4.04)^a^	0.677(±0.111)^a^	0.618(±0.141)^a^
7	11	13.6(±9.07)^a^	0.653(±0.153)^a^	0.606(±0.167)^a^
8	11	12.3(±8.20)^a^	0.629(±0.167)^a^	0.591(±0.175)^a^

Note:

^a^ no significant difference on 0.05 level

### Population structure

**Fig 1:**
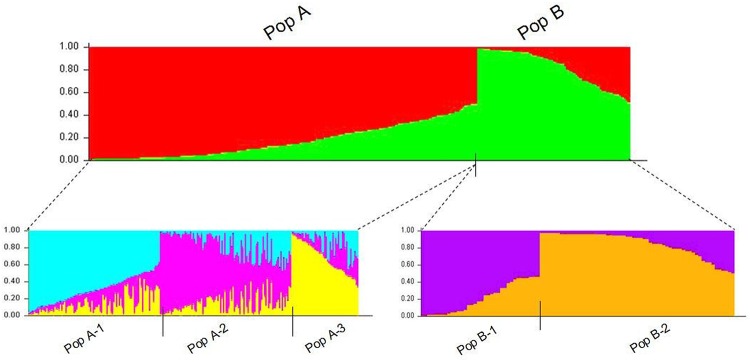
Q-plot showing clustering of 336 tetraploid alfalfa genotypes based on analysis of genotypic data using STRUCTURE.

Analysis of population structure was performed in the complete set of 336 genotypes using the software STRUCTURE. The optimal value of *K* (i.e., the number of clusters) was evaluated by two methods (S2 Fig). Both methods confirmed that the optimal value of *K* was two, i.e., 336 genotypes could be divided into two populations, designed PopA and PopB. PopA included 241 genotypes most from South America, North America, Africa, Europe, West Asia and South Asia. While the Pop B comprised of 95 genotypes most from China. In order to illustrate the two populations, the same analysis were performed within each population respectively, suggesting three subpopulations in PopA, namely PopA-1 (96 genotypes), PopA-2 (96 genotypes), PopA-3 (49 genotypes), and two subpopulations in PopB, namely PopB-1 (36 genotypes), and PopB-2 (59 genotypes) (Fig 1). Clearly, each subpopulation was composed of individuals from accessions from different countries. PopA-1 mainly composed of individuals from Europe (31/44) and North America (38/44), and from Africa (10/28), South America (7/44), West Asia (9/68), and Japan (1/4). PopA-2 mainly included individuals from South America (35/44), Africa (15/28), and South Asia (13/20), and also included some individuals from West Asia (16/68), seven individuals from China, five individuals from Europe, three individuals from Japan, one individual from USA, and one individual from Uzbekistan. PopA-3 mainly composed of individuals from West Asia (32/68), six individuals from Europe (three from Spain, and three from Greece), three individuals from USA, five individuals from Africa, three individuals from Uzbekistan, and two individuals from South America. For PopB, PopB-1 was mainly composed of individuals from China (9/72), Kazakhstan (7/8), Afghanistan (5/12), Turkey (5/28), India (4/12), Pakistan (2/8), Cyprus (1/4), Russia (1/4), and USA (2/28), while PopB-2 mainly from China (56/72), and one individual from each of Pakistan, Kazakhstan, and Russia. Detailed description of membership probabilities of individual accessions is presented in S3 Table. In the present study, the classification of populations appeared rather uncorrelated with the improvement status of the accessions (data not shown).

### Principal coordinate analysis and neighbor-joining based clustering

**Fig 2:**
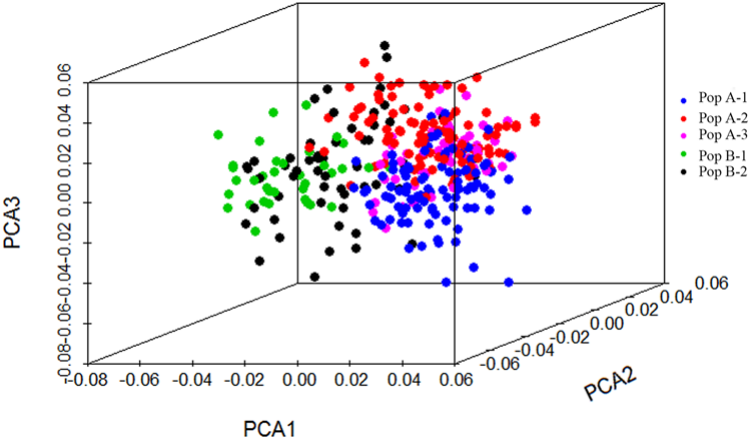
Three-dimensional principal coordinate analysis (PCA) of 336 tetraploid alfalfa genotypes genotyped with SSR markers.

The principal coordinate analysis (PCoA) was conducted to further assess the population subdivisions identified using STRUCTURE. The first three principal coordinates explained 24.2%, 16.4% and 16.0%, respectively, and 56.6% of the total variation. The PCoA was largely consistent with the STRUCTURE analysis, the first principal coordinate (PCo1) clearly separated 336 alfalfa genotypes into two populations as identified by the STRUCTURE analysis (PopA and PopB). The PopB could be divided into two subpopulations (PopB-1 and PopB-2) by the PCo 2, whereas the PopA -1, PopA-2 and PopA-3 were clustered together (Fig 2).

**Fig 3:**
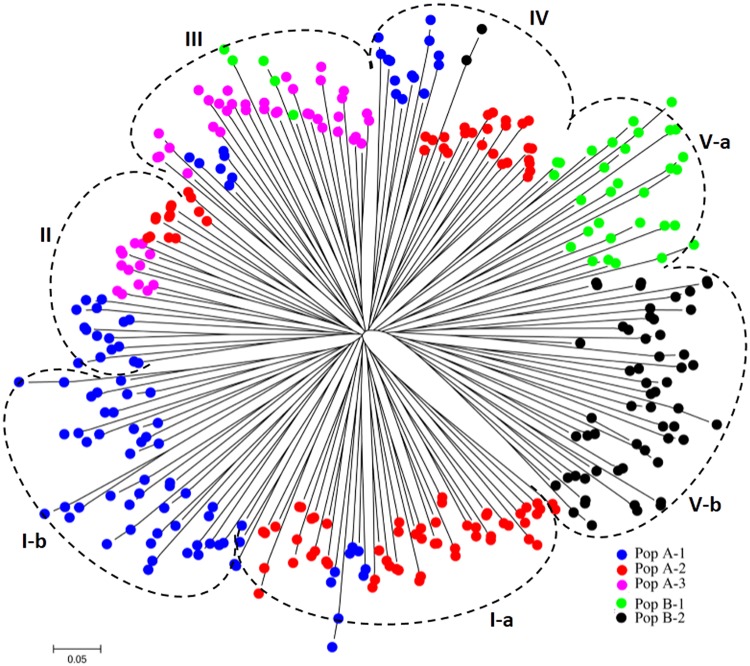
Dendrogram of 336 tetraploid alfalfa genotypes by NJ analysis. Colors in the dendrogram correspond to population structure as identified in structure analysis.

The values of genetic distance (GD) were calculated based on all markers, ranging from 0.09 to 0.59 with the average 0.39 while most of the GD values ranged from 0.35 to 0.45 (ca 76%). Overall, the lowest average GD was observed between wild accessions, followed by landrace (0.391), cultivar (0.387) and cultivated accessions (0.368). No significant difference for the average GD values for different improved types of alfalfa accessions was found. A Neighbor-joining tree of 336 alfalfa genotypes was constructed using Nei’s genetic distance and five clusters I, II, III, IX, and V were identified (Fig 3). Clusters I and V could be further separated into two subclusters namely I-a, I-b, V-a and V-b, respectively. Accessions from Pop A-1 were located mostly in clusters I-b (52.1%), and rest of accessions distributed in I-a, II, III and IV. More than half of individuals from Pop A-2 (58.3%) were in cluster I-a, and 14.6% in cluster II, and 27.1% in cluster IV. Pop A-3 mostly in cluster III (86.5%) with a subset of accessions (13.5%) in cluster II. Pop B-1 mostly in cluster V-a (86.1%) with a subset of accessions (13.5%) in cluster III. Pop B-2 (96.7%) mostly in cluster V-b with two individuals in cluster IV. The clustering of accessions in the unrooted Neighbour Joining tree was generally in agreement with the model-based population structure and PCoA of the collection. The inferred sub-populations were relatively well but not completely separated. Although in some cases individuals from three subpopulations were not grouped together. Still, in most cases they were in the same major population of PopA.

### Genetic diversity and population differentiation

The genetic diversity of each subpopulation identified by the STRUCTURE was assessed (Table 2). PopB-2 enjoyed the highest He and PIC values among 5 subpopulations, which means that alfalfa genotypes from China have high genetic diversity. But no significant difference was found among 5 subpopulations. Among the 1056 alleles detected in the total populations, 201 (19%) were subpopulation-specific. The highest number of population-specific alleles was found in PopA-1 while PopA-3 had the lowest number of population-specific alleles (Table 2).

**Table 2:** Genetic diversity of model-based subpopulations inferred by Structure.

Subpopulations	No. of genotypes	Mean of alleles per locus	Expected Heterozygosity*	PIC	No of population-specific alleles
PopA-1	96	9.6(±4.99)^a^	0.679(±0.135) ^a^	0.638(±0.146) ^a^	55
PopA-2	96	9.5(±5.35) ^a^	0.667(±0.150) ^a^	0.625(±0.158) ^a^	47
PopA-3	49	8.2(±4.23) ^a^	0.672(±0.155) ^a^	0.633(±0.164) ^a^	19
PopB-1	36	8.5(±4.24) ^a^	0.677(±0.150) ^a^	0.638(±0.159) ^a^	40
PopB-2	59	9.0(±4.47) ^a^	0.688(±0.140) ^a^	0.650(±0.150) ^a^	40

Note:

*Based on the assumption of chromosome segregation;

^a^ no significant difference on 0.05 level.

We also determined the genetic diversity for the accessions from cultivars, landraces, cultivated materials, and wild germplasms with different improvement statuses (Table 3). The mean values of alleles per locus ranged from 7.2 for cultivar material to 11.8 for landraces. The same patterns were found for the mean He and PIC values. Landraces had the highest value followed by wild materials and cultivars, while cultivars had the lowest values (Table 3). The three parameters, allele number, the mean expected heterozygosity and PIC values indicated similar distribution patterns as revealed by Violin plots (S3 Fig). Additionally, another two groups were manually sort out on the origins, which were mainly defined as wild (including wild and landrace materials) and cultivated (including cultivars and cultivated materials). The two groups were significantly different in genetic diversity (Table 3). In addition, Analysis of molecular variance analysis (AMOVA) was conducted both in model-based and in improvement status based populations (Table 4). Both of them indicated that there was a much greater proportion of the variation accounted for by differentiation among individuals within populations (>94%), and the remainder (<6%) partitioned among populations (Table 4).

**Table 3:** Genetic diversity of accession groups made according to improvement status.

Improvement status	No. of individuals	Mean of alleles	Expected heterozygosity	PIC
Landraces	156	11.8^a^	0.707^a^	0.673^a^ *
Cultivars	88	10.3^b^	0.684^b^	0.638^b^
Cultivated materials	28	7.2^c^	0.636^b^	0.595^b^
Wild materials	12	6.4^d^	0.680^c^	0.639^c^
Landrace+wild materials	168	11.85^a^	0.696^a^	0.659^a^
Cultivars+cultivated material	116	10.61^b^	0.676^b^	0.637^b^

*The different values with small letters are significantly different at the 0.05 level.

**Table 4:** AMOVA of population differentiation.

	Source of variation	df	Sum of squares	Variance components	Percentage of variation
Model based	Among Pops	4	5.13	0.016	6
	Within Pops	331	81.732	0.247	94
	Total	335	86.862	0.263	100
Improved status based	Among Pops	3	1.940	0.008	4.1
	Within Pops	280	53.499	0.191	95.9
	Total	283	55.439	0.199	100.0

## Discussions

### Genetic diversity of alfalfa germplasm

The study worked on the genetic diversity and population structure of worldwide collection of tetraploid cultivated alfalfa germplasm as accessed by SSR markers. In China, alfalfa has been identified a major forage species for genetic improvement. The genetic diversity revealed by 336 genotypes from a worldwide collection of alfalfa germplasm in the study was high as reflected in average allele number per locus (12.4), He (0.677) and PIC (0.638). Bagavathiannan et al. [14] evaluated the genetic diversity of 12 wild alfalfa populations originating from Canada and 10 alfalfa cultivars and a *M*. *falcata* germplasm using eight SSR markers and identified 16.7 alleles per locus and a He value of 0.77. Flajoulot et al. [13] investigated the diversity level of seven alfalfa cultivars originating from one breeding program with eight SSR markers and found a gene diversity value ranging from 0.665 to 0.717 and 14.9 alleles per locus. The different results could be due to the different populations being evaluated, the marker types used, and the number of markers deployed. When markers common to both sets of studies were compared, the genetic diversity parameters from their studies were lower than our results. For example, our study shared one marker with Flajoulot et al. [13] which detected 17 alleles and He of 0.855 revealed in Flajoulot et al. [13] versus 22 alleles and He of 0.866 in our study. When the genetic diversity of tetraploid cultivated alfalfa was compared to that of diploid germplasm in the *M*. *sativa—falcata* complex, it was slightly lower than that of the diploid germplasm (A = 19.3, He = 0.74, PIC = 0.71) [18]. The similar result was reported by Li et al [33–34] that minimal diversity appeared to have been lost in cultivated tetraploid alfalfa compared to wild diploid alfalfa based on SNP markers. As we know, many alfalfa landraces and plant introductions to China, including even wild diploid germplasm, have been subsequently continuously integrated into cultivated tetraploid breeding germplasm. This germplasm introduction process, together with a relatively short breeding history and the outcrossing and polyploid nature of alfalfa, could explain the high diversity retained in cultivated tetraploid alfalfa [33].

In the present study, alfalfa landraces represented the highest genetic diversity followed by wild materials while cultivars materials had the lowest values (Table 3). One possible reason is that a small number of wild alfalfa accessions (12 individuals) were included in the study compared to the 156 landrace individuals resulted in lower estimates of genetic diversity of wild alfalfa germplasm. Interestingly, the germplasm from China was found to have the highest genetic diversity with the highest values in expected heterozygosity and PIC among five model-based subpopulations in the study, no significant difference was observed among subpopulations though. This experiment revealed the alfalfa germplasm from China had relatively higher genetic diversity than any other places. Alfalfa was initially introduced into China more than 2000 years ago (around 139 BC) [35]. Then more germplasm were introduced via “Land Silk Road” from Iran to West China, from where alfalfa germplasm radiated to other parts of China. Over the long time period natural selection in diverse environments may have generated diverse germplasm as revealed in this study. It cannot be ignored that numerous local cultivars have been selected and formed by farmers. The artificial selection added additional forces to the evolution of alfalfa in China. In recent five decades, Chinese government has introduced modern cultivars from some western countries, which have been used in breeding programs. These factors should be major reasons for the high genetic diversity in the Chinese germplasm used in this study even though the germplasm we used may not fully represent the existing diversity in China.

### Population structure

Population structure analysis enables to understand genetic diversity in a given collection and identify the appropriate population for association mapping [6]. Many previous studies mainly focused on the population structure among the subspecies of the *M*. *sativa—falcata* complex [18, 33]. In the present study, only cultivated tetraploid alfalfa were included to assess the population structure. All cultivated tetraploid alfalfa accessions were consistently separated into two populations by STRUCTURE, PCoA, or cluster analysis (Figs 1, 2 and 3). One is mainly composing of Chinese alfalfa germplasm, the other those alfalfa from other parts of the world. Again different environments, farmers’ selection, geographically isolation, breeders’ selection have contributed to the formation of the unique genetics in Chinese cultivated alfalfa. Although the 336 cultivated tetraploid alfalfa genotypes were divided into two populations and further grouped into five subpopulations in the study, not all the genotypes were grouped together based on the geographical origin. For example, among eight genotypes of accessions CF032020, a Chinese alfalfa cultivar “Zhongmu No 1”, six genotypes were grouped together in Pop B-2, while one genotype was in PopB-1, and another genotype was in popA-2. Alfalfa is an allogamous and heterozygous species. Most cultivars are synthetic populations developed through either phenotypic recurrent selection or natural mass selection with many parents. The recurrent selection methods involving multiple hybridizations and selection activities with available germplasm. One of the parents of “Zhongmu No 1” which was from Peru explains well why one genotype of the cultivar grouped in PopA-2 which was mainly composed germplasm from South America and Africa. Another cultivar “Gannong No.5” (CF037901) characterized by aphid and thrip resistance was separated into three subpopulations, i.e. six genotypes in PopA-2, one in PopB-1, and one in popB-2. This cultivar was bred by three cultivars from Australia SARDI 10, Rippa, and Sceptre. Li et al [33] developed an alfalfa SNP array and used to evaluate patterns of population structure. Within cultivated tetraploid alfalfa, genotypes from dormant and nondormant cultivars were largely assigned to different clusters, while genotypes from semidormant cultivars were split between the groups [33]. In our study, although no clear pattern like that of Li et al. [33] were found, while nondormant genotypes from South America, Africa, South Asia, and West Asia, were grouped together in PopA-2.

It is necessary to use a large number of accessions from each geographical location to confirm whether the clustering pattern of accessions from closer geographical origins agrees with the available pattern identified in this study. However, different relationships may be obtained using different numbers of markers, genome coverage, degree of linkage disequilibrium, the type of DNA sequence variation detected with different marker systems and/or the type of sequence assays (low/high copy)[12].

## Supporting Information

S1 Fig Distribution of average allele numbers (A), Expected heterozygosity (B), and PIC values (C), by chromosome and for all markers. Violin plots show density distribution of PIC values, horizontal bar indicates average value, median is shown as white circle, top and bottom of vertical bar represent the first and third quartile.
(TIF)

S2 Fig A Ln (probability of data) for K ranging from 1 to 10. B Estimating number of subpopulations using delta K values for K ranging from 1 to 10 using method proposed by Evanno et al. (2005)
(TIF)

S3 Fig Distribution of average allele numbers (A), expected heterozygosity (B), and PIC values (C), of accession groups made according to improvement status. Violin plots show density distribution of PIC values, median is shown as white circle, top and bottom of vertical bar represent the first and third quartile.
(TIF)

S1 Table List of materials used in the study. (XLSX)

S2 Table Summary of genetic diversity based on 85 SSR loci of 336 alfalfa genotypes. (XLSX)

S3 Table Membership of alfalfa accessions to clusters as determined by model-based analysis using STRUCTURE. (XLSX)
