# Supplementary figures and images for: Molecular Diversity and Population Structure of a Worldwide Collection of Cultivated Tetraploid Alfalfa (Medicago sativa subsp. sativa L.) Germplasm as Revealed by Microsatellite Markers
Source: PLoS One. 2015 Apr 22;10(4):e0124592. doi: 10.1371/journal.pone.0124592 (PMC4406709; doi:10.1371/journal.pone.0124592)

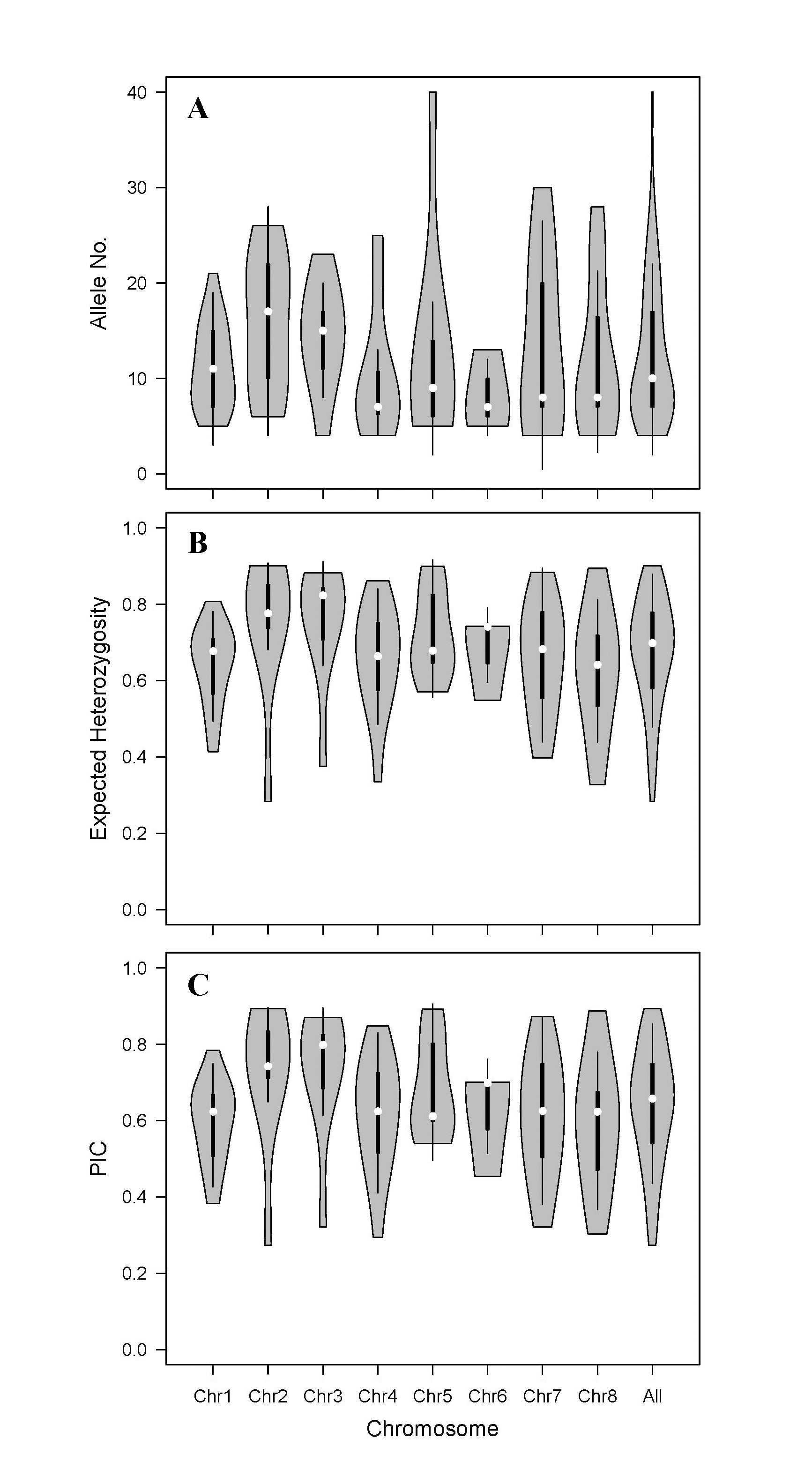

Supplement: S1 Fig — Violin plots show density distribution of PIC values, horizontal bar indicates average value, median is shown as white circle, top and bottom of vertical bar represent the first and third quartile. (TIF) [file pone.0124592.s001.tif]

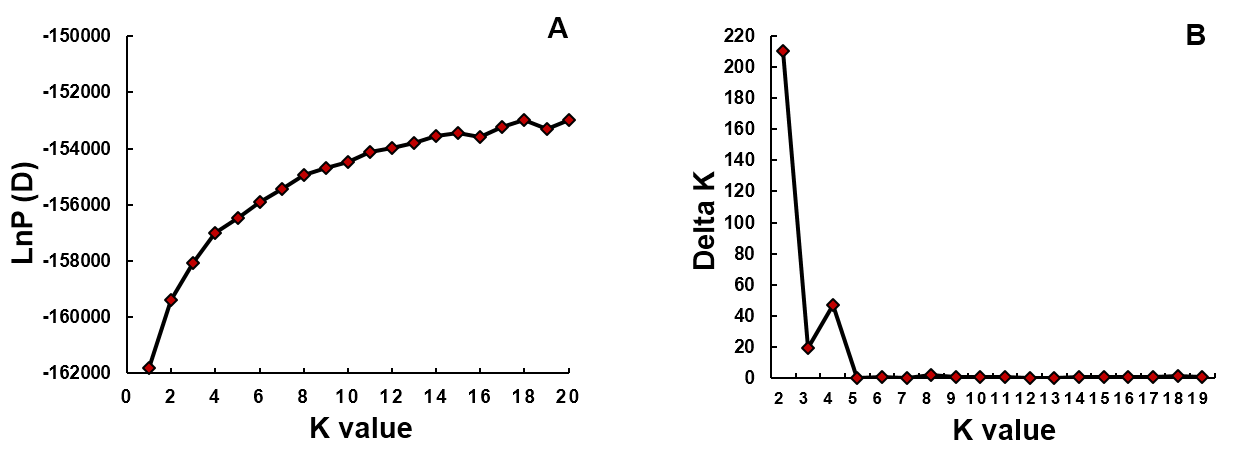

Supplement: S2 Fig — B Estimating number of subpopulations using delta K values for K ranging from 1 to 10 using method proposed by Evanno et al. (2005) (TIF) [file pone.0124592.s002.tif]

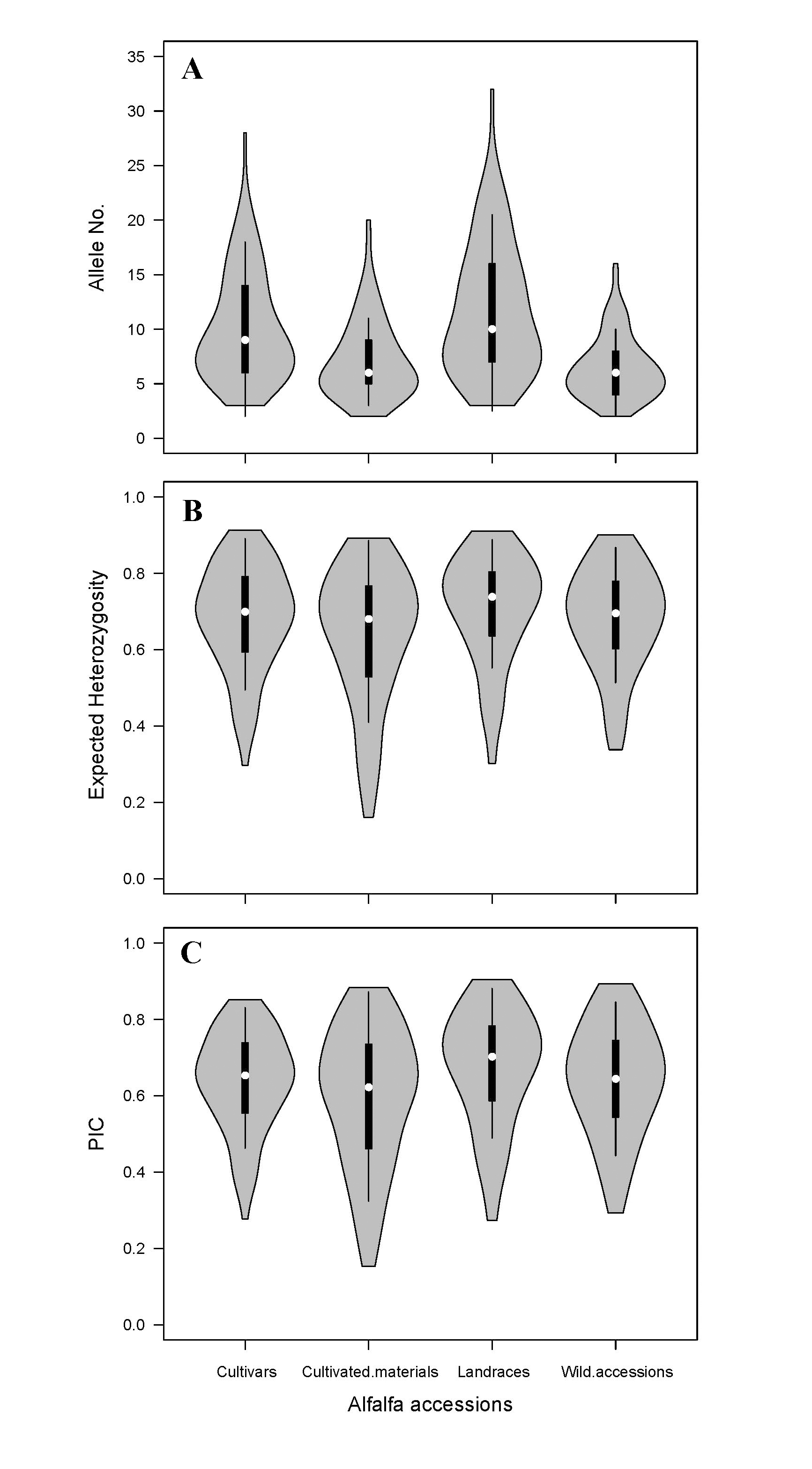

Supplement: S3 Fig — Violin plots show density distribution of PIC values, median is shown as white circle, top and bottom of vertical bar represent the first and third quartile. (TIF) [file pone.0124592.s003.tif]
